# The role of steroids in follicular growth

**DOI:** 10.1186/1477-7827-4-16

**Published:** 2006-04-10

**Authors:** Ann E Drummond

**Affiliations:** 1 Prince Henry's Institute of Medical Research, PO Box 5152, Clayton Victoria 3168, Australia

## Abstract

The steroidogenic pathway within the ovary gives rise to progestins, androgens and oestrogens, all of which act via specific nuclear receptors to regulate reproductive function and maintain fertility. The role of progestins in follicular growth and development is limited, its action confined largely to ovulation, although direct effects on granulosa cell function have been reported. Consistent with these findings, progesterone receptor knockout mice are infertile because they cannot ovulate. Androgens have been shown to promote early follicular growth, but also to impede follicular development by stimulating atresia and apoptosis. The inability of androgens to transduce a signal in mice lacking androgen receptors culminates in reduced fertility. Oestrogens are known to exert effects on granulosa cell growth and differentiation in association with gonadotrophins. Studies with oestrogen receptor knockouts and oestrogen depleted mice have shown us that oestrogen is essential for folliculogenesis beyond the antral stage and is necessary to maintain the female phenotype of ovarian somatic cells. In summary, the action of steroids within the ovary is based on the developmental status of the follicle. In the absence of any single sex steroid, ovarian function and subsequently fertility, are compromised.

## Introduction

Follicular development begins during foetal life with the transformation of primordial germ cells into oocytes and their enclosure in structures called follicles. In most mammals, primordial follicles form either before, or in the first few days after birth. Primordial follicles give rise to primary follicles which transform into preantral (secondary follicles) then antral follicles (tertiary follicles) and finally preovulatory, Graafian follicles, in a co-ordinated series of transitions regulated by hormones and local intraovarian factors. The growth and differentiation of follicles from the primordial population is termed folliculogenesis. With the LH surge, Graafian follicles rupture and oocytes are released, leaving the follicular cells to luteinise and form a corpus luteum.

**Figure 1:**
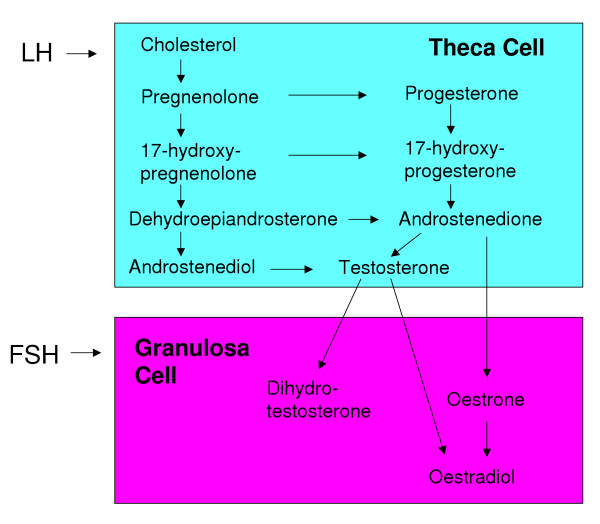
Steroid biosynthesis by the ovary. In the theca, under the influence of LH, cholesterol is converted to pregnenolone and metabolised through a series of substrates ending in androgen production. The two-cell, two-gonadotrophin model comes into play with androgens produced by the theca cells transported to the granulosa cells where they are aromatised to oestrogens.

**Figure 2:**
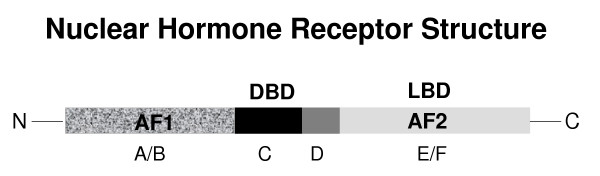
The structure of nuclear hormone receptors. These receptors are composed of 5 structure-function domains labelled A-F (Mangelsdorf et al., 1995) The N-terminal region contains domains A/B, the DNA binding domain (DBD) contains domain C, the hinge region contains domain D and the ligand binding domain (LBD) at the C-terminal end contains domains E/F. Transactivation domains AF1 and AF2 are found in the N-terminal region and the LBD, respectively (White & Parker 1998).

Sex steroids play important roles in the growth and differentiation of reproductive tissues and in the maintenance of fertility. Produced *de novo *from cholesterol, progestins, androgens and oestrogens are synthesised by the ovary in a sequential manner, with each serving as substrate for the subsequent steroid in the pathway. The two-cell, two-gonadotrophin model describes the role of theca and granulosa cells in the production of steroids, highlighting the cooperation between the two cell types, which is necessary for oestrogen production (Figure 1). Given that signal transduction for these hormones usually requires the binding and activation of a ligand-specific receptor, one cannot easily dissociate these components and assign definitive roles. The steroid hormones signal via nuclear receptors to regulate transcriptional events. These receptors form part of a nuclear receptor superfamily, all of which contain common structural elements [1,2]. These include a highly conserved DNA binding domain (DBD), a moderately conserved ligand binding domain (LBD) and 2 transactivation domains, AF1 located in domain A/B and AF2 in domain E/F (Figure 2). This review will address the roles that steroid hormones play in follicular development. It will encompass the direct actions of steroids in the ovary that have been reported and include a discussion of relevant models of ovarian dysfunction and nuclear receptor knockout mouse models that lead to disruption of steroid hormone signalling mechanisms and thus an inability of steroids to fulfil their regulatory roles.

## Progestins

In the female reproductive tract, progesterone plays key roles in ovulation, implantation and the maintenance of pregnancy [3]. This review is confined to the roles progesterone plays in regulating granulosa cell function and follicle rupture during ovulation.

Progesterone receptors (PR) comprise two forms PR-A and PR-B [4,5], although they arise from a single gene. These receptors appear to have functionally different roles [6,7] and *in vitro *data suggests that PR-A is a dominant repressor of PR-B [8,9], with the balance of the two forms determining cellular responsiveness. PR-A and PR-B exhibit different transactivation properties which are cell and promoter specific [5,9-11], indicating actions on separate groups of genes for each receptor, in response to progesterone. They also respond differently to progestin antagonists with PR-B, but not PR-A, becoming a transcriptional activator on binding antiprogestin [12].

PR protein has been localised immunohistochemically to theca of small antral follicles and granulosa cells of preovulatory follicles that had been exposed to LH/hCG, or were undergoing luteinisation, in primate and rabbit ovaries [13,14]. PR mRNA has been localised in immature rat ovaries to granulosa cells of large follicles following PMSG/hCG treatment and to large follicles of adult rat ovaries that had been exposed to the LH surge [15]. When the LH surge was blocked, PR mRNA was not induced. Similarly, LH induced PR mRNA expression by cultured porcine granulosa cells [16]. In cultured rat granulosa cells, PR mRNA was induced by LH in a dose and time dependent manner, with the shorter form, PR-A more abundant than the long form, PR-B [17]. The Antiprogestins RU486 and ZK98299, blocked LH-stimulated luteinisation of granulosa cells isolated from preovulatory follicles [17]. PR mRNA was also expressed by primate granulosa cells following an ovulatory dose of hCG, with whole ovaries and corpora lutea also expressing PR [18]. These findings promote the hypothesis that LH-induced PR is essential for ovulation and luteinisation.

Progestins have been shown to enhance the activity of steroidogenic enzymes in gonadotrophin primed granulosa cells [19] and to enhance progesterone production by these cells [20]. Consistent with this finding, RU486 inhibited progesterone production by cultured human granulosa cells by way of decreasing the activity of 3β hydroxysteroid dehydrogenase [21]. The direct actions of progesterone on granulosa cells extend to: enhancing the response of cultured rat granulosa cells to FSH by increasing cAMP [22]; inhibiting FSH-stimulated oestradiol production by rat granulosa cells [23-25]; inhibiting the primordial to primary follicle transition in newborn rats [26]; inhibiting insulin-dependent granulosa cell mitosis [27,28] and inhibiting apoptosis by large granulosa cells in culture (granulosa cells isolated from immature rat ovaries were separated based on their size eg small or large), which do not express PR [29]. It was puzzling that RU486, a PR antagonist, inhibited the anti-apoptotic action of progesterone, given the absence of receptors from granulosa cell. Consequently it has been proposed that some of the actions of progesterone, notably the inhibition of apoptosis and insulin-dependent mitosis, are mediated by non-genomic mechanisms [30,31]. The identification of a progesterone binding protein (60 kDa P_4_BP) within ovarian and granulosa cell lysates [32] and progestin membrane receptors in rat corpora lutea [33], a tissue that does not express detectable levels of nuclear progesterone receptors, may facilitate the actions of progesterone.

At ovulation, the Graafian follicle ruptures releasing the ovum. Thereafter, the follicle undergoes remodelling to form a corpus luteum, which secretes progesterone. The ovulatory process is progesterone dependent. Inhibitors of progesterone synthesis, notably aminoglutethimide, epostane and RU486, have been shown to block LH-induced ovulation *in vivo *and *in vitro *in rats and mice [34-37]. There is also the suggestion that progesterone is involved in stimulating ovulation in the human [38]. Firstly, it has been shown that the rise in progesterone precedes that of gonadotrophins [39,40]. Secondly, in luteinised unruptured follicle (LUF) syndrome, the preovulatory rise in progesterone observed in normal women is delayed and follicles fail to rupture although they undergo luteinisation [41]. Thirdly, the administration of RU486 blocked the LH surge and ovulation [42]. In specific terms, progesterone has been shown to enhance the production of proteolytic enzymes important for the rupture of follicles at ovulation [43], either directly, or by enhancing endometrial relaxin production [44], which is thought to stimulate the release of proteases by granulosa cells [45]. The expression of two metalloproteinases ADAMTS-1 and cathepsin-L, thought to be required for ovulation, by granulosa cells of anovulatory follicles of PR null mice, is inhibited indicating a dependence on PR for normal expression [46].

The importance of PR in female reproduction is underscored by the infertility of PR null mice [47]. Despite histologically normal ovaries, these mice fail to ovulate even after exogenous stimulation with gonadotrophins, as indicated by the presence of unruptured preovulatory follicles in the ovary and an absence of oocytes in the oviduct and uterine horns [47]. The granulosa cells of these preovulatory follicles luteinise, suggesting that PR is not required for corpus luteum formation [46]. However, this observation conflicts with the credible report of Natraj and Richards (1993) [17], that progesterone acting at least in part via PR, is required for luteinisation (see earlier paragraph). The development of PR-A and PR-B knockouts reveals that PR-A is necessary for ovulation whereas PR-B is not [48,49]. Consequently, PR-A knockouts were infertile, the result of a failure to ovulate, whereas PR-B knockouts were ovulatory and produced viable offspring. These studies further highlight the capacity of PR-A and PR-B to mediate different actions of progesterone [50].

## Androgens

Androgens, primarily androstenedione and testosterone, are produced by theca cells in response to LH (Figure 1). Androgens act via receptors (AR) localised to granulosa cells, stromal cells [51-53], human theca cells [54] and more recently, to rat, pig and mouse oocytes [55-57]. Androgens predominantly target granulosa cells where they initiate scenarios depending on the developmental status of the follicle and species. AR expression is highest in granulosa cells of: preantral and antral follicles of primate ovaries [58,59]; small preantral and early antral follicles of rat ovaries [53,60]; small antral follicles of porcine ovaries [61]; preantral to early antral follicles of bovine ovaries [62] and secondary and dominant follicles of human ovaries [54]. The expression of AR may be influenced by oocyte secreted factors. Tetsuka and colleagues [53] reported that a gradient of AR immunostaining existed in large follicles of the rat ovary, with cumulus cells and antral granulosa cells strongly expressing AR protein and peripheral layers expressing less [53].

In the early stages of folliculogenesis, androgens appear to promote follicular growth. Administration of testosterone to pregnant ewes and thus prenatal treatment of fetuses with androgen, led to decreased numbers of primordial follicles and enhanced numbers of follicles at other stages of development, indicating that testosterone was enhancing follicular recruitment [63]. Since testosterone can be aromatised to oestrogen at this stage of development, it is difficult to determine whether this is a direct effect of testosterone or an indirect effect, by way of providing substrate for aromatisation. In female rhesus monkeys prenatally androgenised with testosterone, large polyfollicular ovaries that resemble polycystic ovaries found in women with polycystic ovary syndrome (PCOS) develop [64-66]. The diameter of mouse preantral follicles cultured *in vitro *for four days with either testosterone, dihydrotestosterone, androstenedione, dehydroepiandrosterone or dehydroepiandrosterone sulfate, expanded and the granulosa cells labelled strongly with bromodeoxyuridine, an indicator of cellular proliferation [67]. An AR antagonist, but not an aromatase inhibitor, inhibited the growth response, indicating that oestrogen converted from the androgens in culture, was not responsible for follicle growth. A direct stimulatory effect of androgens on mouse antral follicle growth *in vitro*, has also been demonstrated [68]. More recently, dihydrotestosterone has been shown to enhance porcine granulosa cell proliferation, by potentiating the effects of factors, most likely GDF9, secreted by denuded oocytes *in vitro *[69]. In primates treated short term with androgens, increased numbers of preantral and small antral follicles were present in the ovary and the theca had undergone hypertrophy [70]. In pigs, numbers of preovulatory follicles and corpora lutea were increased when treated with testosterone or dihydrotestosterone during the follicular phase [56,71,72]. During late preovulatory development, AR decline in most mammals, the exception being humans and androgens are metabolised as opposed to exerting direct effects on folliculogenesis [73].

Apart from effects on growth, androgens have been shown to enhance the follicle stimulating hormone (FSH)-mediated differentiation of granulosa cells, as indicated by an increase in progesterone and oestradiol production [74-76] and to play roles in oocyte maturation. *In vitro*, testosterone induced the maturation of mouse oocytes arrested at meiosis, via a transcription independent mechanism [57]. The addition of flutamide, an AR antagonist, blocked the maturation. Stimulatory effects of dihydrotestosterone on FSH receptor mRNA expression by preovulatory follicles of gilts [56] and testosterone on FSH receptor mRNA expression by primate primary follicles [77], have also been reported. These studies suggest that androgens can facilitate the response of follicles to FSH. Components of the ovarian IGF-I signalling system have been shown to be regulated by androgens. IGF-I and IGF-I receptor mRNAs have been shown to be enhanced by both testosterone and dihydrotestosterone in primates [78,79]. The broad localisation of the IGF-I and IGF-I receptor mRNA's (granulosa cells, theca, oocytes and interstitial cells) suggests wide ranging roles for androgen in ovarian function.

Androgens have also been shown to impede follicular development, enhancing follicular atresia in immature rats primed with PMSG [80,81] and in estrogen-treated hypophysectomised immature rats [82]. A single injection of dihydrotestosterone to cycling mice reduced the number of large follicles by fifty percent [83] and left the mice subfertile. Androgens are reported to inhibit FSH-stimulated LH receptor expression by granulosa cells [84,85] and to modulate granulosa cell apoptosis, enhancing the process in the rat [86], but demonstrating a negative correlation with androgen receptor suggestive of reduced effects on granulosa cell apoptosis, in the rhesus monkey [58].

In humans, hyperandrogenism is a classic symptom of PCOS [Reviews: [87-89]]. Abnormal steroidogenesis by the ovary is responsible for the androgen excess, which is thought to impact on the level and distribution of adiposity in PCOS patients and predispose them to insulin resistance and anovulation [90]. It is thought that the theca cells of PCOS ovaries are not responsive to downregulation by gonadotrophins allowing for unchecked androgen production [91]. The hypertrophy of theca, which occurs in PCOS ovaries, exacerbates the problem. Evidence for the role of androgens in anovulation comes from PCOS patients treated with the antiandrogen, flutamide. After six months treatment, ovulation was restored [92]. In addition, when hormone therapy for ovulation induction (clomiphene and follicle stimulating hormone regimen) was supplemented with cyproterone acetate, an antiandrogen, ovulation and pregnancy rates were highest [93]. These reports however, are counterbalanced by a study in which the antiandrogen, nilutamide, did not stimulate ovulation in anovulatory PCOS patients [94]. The cystic nature of PCOS ovaries has been replicated in the immature rat with a view to determining why anovulation occurs. Collagen degradation of the follicular wall at ovulation is essential for oocyte release. The role of matrix metalloproteinases (MMP), which degrade collagen and lysyl oxidase (LOX), a cross-linker of collagen and elastin normally involved in collagen repair or reconstruction, were investigated in this dehydroepiandrosterone-induced rat model of PCOS [95]. The study established that in response to androgen, MMP2 activity was significantly reduced whereas LOX activity was significantly enhanced, indicating that collagen breakdown and follicle rupture could potentially be inhibited, thus explaining the cystogenesis that occurs in PCOS. Clearly, the mechanisms that lead to anovulation in a sub group of PCOS patients are complex and not fully understood. Recent criteria defining PCOS indicate that the ovary should contain 12 follicles or more, 2–9 mm in diameter, or ovarian volume greater than 10 cm^3 ^[96]. The premature development of follicles in PCOS ovaries is thought to be due to the elevated levels of androgen. Support for this hypothesis comes from the primate study discussed earlier [70]. Follicle development is either arrested as in anovulatory PCOS, the follicles remaining healthy and steroidogenic [97], or tending toward atresia as in ovulatory PCOS [98]. Elevated levels of androgens are thought to activate cell death pathways in preantral granulosa cells [87]. The low levels of aromatase in atretic follicles foster the conversion of androgens to dihydrotestosterone rather than to oestrogen, perpetuating the androgen excess [99,100]. Recent studies by Maciel and colleagues [101] propose an alternative theory to androgen stimulated follicular growth; that follicular growth is actually slowed in PCOS ovaries causing primary follicles to 'stockpile' [101]. Significantly more primary, secondary and Graafian follicles were found in PCOS ovaries compared with normal ovaries, although the greatest increase in follicle numbers was observed in what was termed 'classic' primary follicles, that is those follicles in which the oocyte is surrounded by a single layer of fully cuboidal granulosa cells. No changes in primordial follicle numbers or the numbers of atretic follicles were seen in PCOS ovaries [101], adding weight to the 'stockpiling' theory.

Despite the apparent roles for androgens in the ovary, studies on the testicular feminised mouse (Tfm), which lack functional androgen receptors, indicate that AR are not essential for fertility [102]. The reproductive lifespan of these mice however, was reduced due to accelerated aging of the ovary and they were subfertile [102]. The development of an androgen receptor conditional knockout has furthered studies on androgen action in the ovary. Female AR knockout mice have longer oestrous cycles and reduced fertility, evident in fewer litters and reduced numbers of pups. Their ovaries contain normal numbers of follicles, although large antral follicles appeared to have fewer granulosa cells and there were reduced numbers of corpora lutea [103]. These observations are consistent with studies in PMSG treated immature rats, where androgen reduced ovulation rate by decreasing the number of granulosa cells per follicle [81]. Superovulation induction in these androgen receptor knockouts, led to the formation of abnormal cumulus-oocyte complexes [103].

One of the most important roles played by androgens in the ovary is in the synthesis of oestrogen. Androgens serve as substrates of P450 aromatase, which mediates the conversion to oestrogens [[104], Reviews [73,105]].

## Oestrogens

The capacity of follicles to make oestrogen is first apparent in the late preantral stage when they possess all the components of the 'two cell, two gonadotrophins' model. Although aromatase activity is present in small antral follicles, oestrogen production at this stage of development is limited by an inability to produce androgen substrate for aromatization to oestrogen [106]. Growth beyond the small antral stage is therefore characterised by increased aromatase activity and androgen synthesis, which culminates in follicular oestrogen production. The preovulatory follicle has the highest intrafollicular levels of estradiol, primarily due to the size of its granulosa cell population and its capacity for androgen aromatisation [107,108].

Oestrogens signal via receptors (ER) of which there are two forms, ERα and ERβ [109-113], with ERβ being the predominant form in the ovary [114,115]. A number of variant forms of ERβ have been identified in the human [Review: [116]]. ERβ mRNAs lacking exon 5 and/or 6 [117] and ERβ2/cx, ERβ2, ERβ4 and ERβ5 [118,119] mRNAs are all expressed by the ovary. The functional significance of these forms however, is unclear. During postnatal development, the mRNA expression of ERβ increases in synergy with the proliferation of granulosa cells in the rat ovary. ERα mRNA levels in contrast, remain stable after its initial induction [115], indicative of a more widespread expression profile as highlighted by protein localisation studies in the ovary [120-122]. Recent studies using isotype-selective, estrogen receptor agonists have assessed the individual roles that ERα and ERβ play in female reproduction [123]. Distinct roles for each receptor were identified: ERα inhibited ovulation, most likely via an effect on the hypothalamo-pituitary axis and uterine growth; while ERβ stimulated follicular growth, decreased atresia, induced the expression of specific genes and enhanced the number of oocytes released following ovulation induction.

Oestrogen has acknowledged local intrafollicular actions [[124,125], Reviews: [126-128]]. Administration of oestrogen to hypophysectomised rats stimulates the proliferation of granulosa cells in small preantral follicles and reduces atresia [129,130]. Subsequent administration of FSH to these oestrogen-primed hypophysectomised rats results in increased follicular growth and differentiation and antrum formation [131]. It is interesting to note that although oestrogen alone is a potent mitogen on rodent granulosa cells *in vivo*, it is devoid of mitogenic activity *in vitro*. This indicates that the *in vitro *culture systems are incapable of maintaining the mitogenic responsiveness of granulosa cells to oestrogen and/or other intrafollicular growth factors, some of thecal origin, may be involved in the mitogenic actions of oestrogen [132]. Studies of Bley and colleagues (1997) [133] indicate that a combination of oestrogen and FSH or androgen, perhaps aromatised *in vitro *to oestrogen and FSH, can stimulate granulosa cell proliferation *in vitro *and that this effect is further amplified by insulin or IGF-1 [133]. It is not clear what genes oestrogen induces in granulosa cells, but cyclin D2 [134], inhibin α and inhibin βB [135] are likely candidates.

Oestrogen is also responsible for facilitating the differentiation of granulosa cells including the induction of receptor systems for FSH, LH and prolactin and it can influence post-receptor mechanisms. In conjunction with LH and FSH, E_2 _stimulates cAMP accumulation [136] and increases the number of cAMP binding sites in granulosa cells [137]. There is now evidence that FSH can activate the IGF-1/phosphatidylinositol 3-kinase (PI3K)/phosphatidylinositol-dependent kinase 1 (PDK1) pathway in granulosa cells with serum and glucocorticoid-induced kinase (Sgk) and protein kinase B (PKB) or Akt, kinases being phosphorylated [138]. These effects can be mimicked by forskolin, cAMP and IGF-1 and can conceivably be enhanced by oestrogen. Targets for PKB and Sgk include members of the forkhead (FOX) family of transcription factors of which forkhead homologue of rhabdomysarcoma (FKHR) is a member. FKHR is expressed by granulosa cells, its expression enhanced by FSH and oestrogen [138]. The role of FKHR in granulosa cells has yet to be elucidated but it may be linked with proliferation since cells expressing FKHR also expressed cyclin D2 and showed enhanced staining for proliferating cell nuclear antigen (PCNA) [139,140].

### Oestrogen Receptor Knockout mice

In recent years, oestrogen receptor knockout mice have been generated in an effort to define the points of oestrogen action in the ovary. The ERα knockout (ERKO) and ERβ knockout (BERKO) mice however, are not oestrogen-free given their capacity to transduce a signal via the alternative oestrogen receptor subtype. The female ERKOs are acyclic, infertile and possess hyperemic ovaries devoid of corpora lutea. Folliculogenesis is arrested at the antral stage with large follicles becoming cystic and haemorrhagic [141,142]. Prolonged administration of a GnRH antagonist to ERKO mice prevented formation of the haemorrhagic cysts [143], indicating that the ovarian phenotype manifests as a consequence of elevated LH levels [144,145]. Gonadotrophin-treated prepubescent ERKO mice ovulate, although they released fewer oocytes compared to their wildtype counterparts [146]. Thus, it would appear that folliculogenesis, ovulation and corpora lutea formation can occur in the absence of ERα, albeit suboptimally.

The female BERKOs [147-149] have small ovaries, partially arrested follicular development associated with increased numbers of primordial follicles, but significantly fewer numbers of primary and large antral follicles and corpora lutea. Increased atresia of large follicles is evident. In culture, BERKO follicle growth was retarded and they produced significantly less oestrogen and expressed less aromatase than wildtype follicles [149]. Compromised fertility was evident in reduced numbers of offspring/litter, consistent with the reduced numbers of corpora lutea [147]. Following ovulation induction, fewer oocytes were released from BERKO ovaries relative to their wildtype counterparts. This is likely to be due to reduced Ptgs2 expression, a gene necessary for ovulation, by BERKO follicles after gonadotrophin stimulation [149,150]. Interestingly, gonadotrophin levels in these mice are normal. These studies indicate that ERβ plays an essential role in gonadotrophin-induced granulosa cell differentiation. In its absence, follicle maturation and the ovulatory process are impaired. Androgen receptor (AR) analyses of BERKO mice ovaries revealed an increased expression of AR in late antral and atretic follicles [148], a time in wildtype animals when AR expression is low. Androgens are known to enhance atresia [80-82] and thus the overexpression of AR by the ovaries of BERKO mice most likely accounts for the increased atresia and premature exhaustion of follicles observed in these mice. Treatment with the antiandrogen, flutamide reversed the BERKO ovarian phenotype, the ovaries containing healthy late antral follicles and increased numbers of corpora lutea [148]. A role for androgens in the manifestation of the BERKO phenotype and thus in ovarian folliculogenesis is clearly indicated.

The generation of double ER knockout (αβERKO) mice by two laboratories [151,152] and reports of their ovarian phenotype, indicate that these mice are distinct from the individual ER knockouts. These ovaries exhibit follicular trans/re-differentiation with tubular-like structures containing Sertoli-like cells. Where oocytes were present these were seen to be degenerating, raising the possibility that factors produced by the oocyte may be involved in the transformation process. The capacity to be effected by oestrogens or a lack there of, is asserted by the presence of ERα mRNA in mouse and human oocytes [153-155]. The phenotype is expressed in the presence of elevated LH levels, similar to that of the ERKO mouse. Thus it appears that both ERs have roles to play in the maintenance of fertility, although ERβ appears essential for follicle development and maturation.

### Oestrogen depletion – the ArKO mouse

An alternative model to ER depletion is to remove oestrogen itself from the ovary. Targeted deletion of most of exon 9 of the *Cyp 19 *(aromatase) gene has given rise to a mouse which lacks functional aromatase and thus an inability to produce oestrogen [156]. Female ArKO mice have undetectable levels of aromatase and oestrogens but exhibit high levels of testosterone, FSH and LH in serum. These reproductive hormones have been implicated as playing crucial roles in various aspects of folliculogenesis [Reviews: [157,158]]. The ArKO model has allowed us to define how far follicles can grow in the total absence of oestrogen [156,159,160]. We established that follicle development was arrested at the antral stage rendering these mice infertile due to an inability to ovulate. The antral follicles that were present appeared morphologically atretic or prematurely luteinized, as evidenced by the presence of pyknotic nuclei or cytoplasmic lipid droplets, respectively. The phenotype exacerbates with age, the ovarian interstitium becoming increasingly diffuse and containing increasing numbers of morphologically abnormal follicles and haemorraghic cysts [160]. Secondary and antral follicles become less common in the ovary and eventually the number of primary follicles also decreases [159,161]. Recent studies quantitating the primordial follicle pool, indicate that there are reduced numbers of primordial follicles in ArKO ovaries and that their oocytes are enlarged [162].

The phenotype of the somatic cells in the ArKO ovary, has been investigated both ultrastructurally and immunohistochemically [160,161] and gene expression studies have been performed [163]. Detailed light microscopy identified the abnormal follicles as seminiferous tubule-like structures filled with Sertoli-like somatic cells, apparently arising from the trans/re-differentiation of granulosa cells. These Sertoli-like cells possess adult-type Sertoli cells characteristics, including a tall irregular columnar shape and lateral cylindrical-like processes; basally located nuclei; prominent tripartite nucleoli and a homogeneous nuclear chromatin distribution, specialised desmosome-like adherens junctions and Sertoli-cell specific, ectoplasmic specialisations between cells [160]. Cells morphologically resembling testicular Leydig cells were present within the interstitial regions of ArKO ovaries [160,164]. These Leydig cell-like cells contain an abundance of smooth endoplasmic reticulum, often present in whorl-like formations and the nucleus accommodated an annular nucleolus, all characteristics of murine Leydig cells. It is unclear at this stage if these cells are functional ie., whether they possess steroidogenic enzymes. The expression of the male type genes, Sox 9 and Mullerian inhibiting substance (MIS) were increased in ArKO mice ovaries [163], indicating that oestrogen is required for maintenance of the female phenotype of ovarian somatic cells. Thus, in the absence of oestrogen, the ovaries undergo sex reversal and testicular type cells appear, indicating that gonadal cells retain a degree of plasticity. Oestrogen replacement partially restored the ovarian phenotype, eliminating male-like cells from the ovary and allowing limited ovulation in some ArKO mice [162,163].

These data confirm and extend observations in αβERKO mice in which Sertoli-like cells with similar properties were observed in the ovaries [151,152]. These compound ER knockouts are not oestrogen-free and it is possible that there remains an influence of oestrogen via some as-yet unidentified ER, particularly since an ERγ form has been identified in fish [165]. A non-genomic action of oestrogen can also not be ruled out. The presence of Sertoli-like and Leydig-like cells in the ovaries of ArKO mice on a phytoestrogen-free diet, indicates that these cells only develop in the complete absence of oestrogen. Thus oestrogen is required for normal folliculogenesis, from the antral stage on. The development of antral and preovulatory follicles is prerequisite for ovulation and corpus luteum formation and for the maintenance of fertility.

The role of the oocyte in the trans/redifferentiation of granulosa cells in these animal models has been unclear. Recent studies in irradiated rats [166] have addressed this question. Oocytes in non-growing follicles were selectively destroyed by gamma irradiation. The follicular cells that remained differentiated into Sertoli-like cells and expressed morphological characteristics of Sertoli cells, although some traits of follicular cells, such as the expression of FOXL2 protein remained. These cells did not express oestrogen receptors, so despite normal levels of oestrogen, a signal could not be transduced by them. Despite similarities with the sex reversed cells reported in ArKO or ER double knockout mouse ovaries, these transdifferentiated follicular cells did not express Sox 9. They also established a role for FSH in the development of these transdifferentiated oocyte depleted follicles. While FSH levels are elevated in ArKO mice [159], a role for FSH in the development of sex-reversed cells has yet to be shown. Studies to elucidate the oestrogen-mediated mechanisms that operate between the oocyte and the somatic cells, to maintain and promote the follicular-granulosa cell phenotype are only just starting to be undertaken. Otsuka and colleagues [167] recently reported that oocytes mediate oestrogen's enhancement of FSH action on P450 aromatase, FSH and LH receptor and inhibin/activin subunit mRNA expression and cAMP production by granulosa cells *in vitro *[167].

## Conclusion

Steroid hormones, via ligand-specific receptors, play important regulatory roles in the ovary. The impact of these hormones on ovarian function is determined by ligand availability, receptor expression and the repression or induction of relevant regulatory genes. Ultimately, it is the needs of individual follicle populations, which determine the roles that steroids play. From the data presented in this review, it is clear that each steroid plays roles of consequence for fertility. In their absence, or in cases of excess, ovarian function and subsequently fertility, is compromised.
